# Research Progress on Separation and Extraction Technologies of Lignin

**DOI:** 10.3390/ma19101998

**Published:** 2026-05-12

**Authors:** Dingkai Wang, Mingyu Cui, Xutang Liu, Meiling Liu, Xiaopeng Han, Xiaoming Xiong, Shanglong Chen, Shangshang Ma, Qiqi Sun, Lingfeng Jiao, Wei Zhao

**Affiliations:** 1 College of Chemical Engineering, Shanxi Institute of Science and Technology, Jincheng 048000, Chinasunqiqi@sxist.edu.cn (Q.S.); jiaolingfeng@sxist.edu.cn (L.J.); 2 State Key Laboratory of Materials Processing and Die & Mould Technology, School of Materials Science and Engineering, Huazhong University of Science and Technology, Wuhan 430074, China; 3 Jiangxi Provincial Key Laboratory of Flexible Electronics, Flexible Electronics Innovation Institute, Jiangxi Science and Technology Normal University, Nanchang 330013, China; 4 Jiangsu Key Construction Laboratory of Food Resources Development and Quality Safe, College of Food and Biological Engineering, Xuzhou University of Technology, Xuzhou 221018, China; slchen1982@163.com; 5 Key Laboratory of Spin Electron and Nanomaterials of Anhui Higher Education Institutes, School of Chemistry and Chemical Engineering, Suzhou University, Suzhou 234000, China; szxymashangshang@163.com; 6 Key Laboratory of Coal Processing and Efficient Utilization, China University of Mining & Technology, Xuzhou 221116, China

**Keywords:** lignocellulose, lignin, extraction, organosolv, ionic liquids

## Abstract

Lignin, a complex natural three-dimensional aromatic polymer, is prone to condensation during the separation process, owing to the diverse properties of its basic structural units, linkage types, and spatial configurations. These inherent structural complexities present significant challenges for its efficient isolation and precise transformation. Current separation techniques primarily include physical, chemical (such as acid hydrolysis, alkaline dissolution, organic solvents, and ionic liquids), and biological methods. Each approach offers distinct advantages and limitations in terms of yield, purity, cost, and impact on lignin structure. Studies have indicated that ionic liquids and organic solvent methods demonstrate considerable application potential owing to their mild reaction conditions and high selectivity. Future research should focus on developing green, efficient, and low-cost separation technologies, while also enhancing detailed structural characterization and targeted lignin conversion to facilitate its large-scale utilization in the production of value-added materials and chemicals.

## 1. Introduction

**Figure 1:**
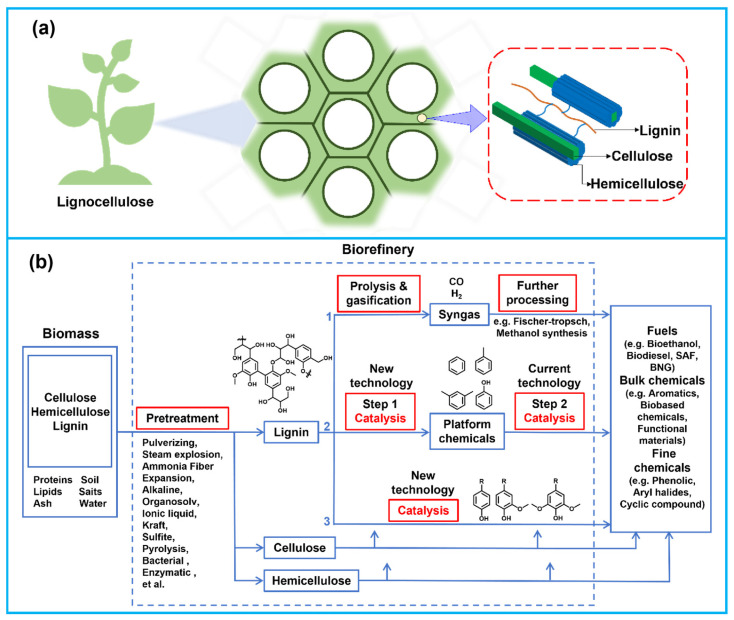
(**a**) Composition of lignocellulosic biomass, (**b**) biorefinery of lignocellulose feedstocks [3,4,5]. Copyright 2010, American Chemical Society.

The outer layer of lignocellulose is encased by a highly complex, three-dimensionally cross-linked amorphous resin (lignin). This structure not only protects internal carbohydrates and proteins from microbial attack and water damage but also confers structural integrity and rigidity (Figure 1a) [1]. However, the protective role of lignin concurrently elevates the difficulty in valorizing the primary internal components (cellulose and hemicellulose) [2]. To enhance the full-component utilization of biomass, effective pretreatment of lignocellulose is therefore required to separate the lignin barrier. In recent years, with the advancement of biomass refining, the utilization of lignocellulose has transitioned from single-component extraction to all-component separation and conversion, as illustrated in Figure 1b. Thus, it is necessary to design novel separation technologies by integrating the structural properties of raw materials with value-added products.

In terms of feedstock properties, lignocellulosic fibers display insufficient length and inferior mechanical strength, rendering them unsuitable for cellulose-based materials requiring high mechanical performance. If the target products are small-molecule compounds (liquid fuels and chemicals), it is necessary not only to maximize component separation but also to construct a sugar platform [6]. Compared with fiber components, lignin inherently possesses a complex structure, while its separation is often accompanied by the cleavage of aryl ether bonds and the formation of C-C condensed linkages, ultimately leading to the collapsed framework and diminished reactivity. Under existing industrial conditions, the efficient separation and value-added conversion of lignin remain elusive [7,8]. Nevertheless, as the sole renewable aromatic polymer in nature, lignin holds substantial promise for the production of fuels and aromatic chemicals, underscoring its wide-ranging application prospects [9]. Therefore, the separation and extraction of lignin have emerged as a prominent research focus, and how to effectively separate the three major components remains a critical challenge for achieving value-added utilization of biomass.

## 2. Structure of Lignin Materials

### 2.1. Basic Structural Units of Lignin

**Figure 2:**
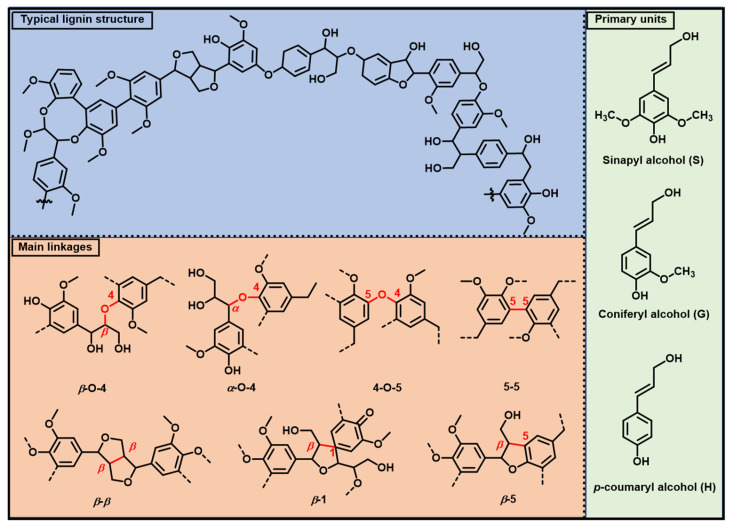
Basic structural units of lignin and their main linkages [12,13]. Copyright 2022, Royal Society of Chemistry.

In lignocellulose, lignin is primarily a complex, three-dimensionally cross-linked macromolecule that confers strength and rigidity to plant cell walls. Currently, the composition and content of lignin in plants exhibit variation with respect to plant type and distribution. Lignin primarily consists of three basic structural units: sinapyl alcohol (3,5-dimethoxy-4-hydroxycinnamyl alcohol), coniferyl alcohol (3-methoxy-4-hydroxycinnamyl alcohol), and *p*-coumaryl alcohol (4-hydroxycinnamyl alcohol). These three monomers, commonly designated as S-units, G-units, and H-units, respectively (Figure 2), share a unifying phenylpropane-based structural feature. The distinction among these monomers resides in their methoxy content (sinapyl alcohol with two, coniferyl alcohol with one, and *p*-coumaryl alcohol with none), and their respective contents correlate closely with lignin type. For instance, softwood (gymnosperm) lignin is predominantly rich in G-units, hardwood (angiosperm) lignin comprises both G and S units, and grass lignin is a mixture of H, G, and S units [10]. Depending on the relative abundance of its basic structural units, lignin is categorized into G-type (softwood lignin), G-S type (hardwood lignin), H-G-S type (herbaceous lignin), and H-G type (compressed wood lignin) [11].

### 2.2. Main Linkages of Lignin

Lignin is primarily formed by the coupling of three monomers via C-O and C-C bonds (Figure 2). In natural lignin, C-O bonds account for over two-thirds of total linkages, followed by C-C bonds [14]. To classify the various linkages, aliphatic side-chain carbons are labeled *α*, *β*, and *γ*, while those in aromatic rings are labeled 1–6. For instance, a *β*-O-4 bond is the linkage between the *b*-carbon of the aliphatic side chain and the oxygen atom attached to C_4_ of the aromatic ring. Additionally, there are also other linkages, including *β*-*β*, *β*-5, 4-O-5, 5-5, *α*-O-*γ*, and *β*-1, etc. Notably, even for the same plant species, these data can vary due to growing environment, geographical location, and even analytical methods.

### 2.3. Spatial Structure of Lignin

Although the basic structural units of lignin and their inter-unit linkages have been characterized, the randomness and unpredictability of these linkages pose challenges to understanding its molecular structure. Studies have shown that the random polymeric nature and three-dimensionally folded architecture of lignin inevitably trigger repolymerization, self-condensation, and ultimately coking. Furthermore, free radicals and C-C bond self-condensation reactions tend to occur during the reaction [15,16]. Using density functional theory, Qi et al. [17] simulated a small lignin fragment (SLF, C_78_H_90_O_25_) containing 8 monomer units and a large lignin fragment (BLF, C_188_H_214_O_66_) containing 19 monomer units. The overall structural relaxation of SLF and BLF assumes a folded geometry, and moreover, their strong intramolecular hydrogen bonds facilitate folding and contraction. This not only renders lignin depolymerization more challenging but also impedes understanding of its actual molecular structure.

## 3. Strategies for Lignin Separation and Extraction

**Figure 3:**
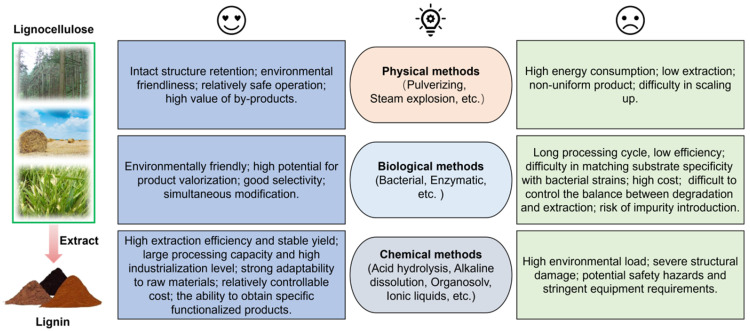
The primary strategies for lignin separation and extraction [18,19].

Currently, the primary strategies for lignin separation include physical, chemical, and biological methods [18,19,20], with detailed pretreatment procedures summarized in Figure 3. The physical method entails disrupting the linkages between lignin and hemicellulose via steam explosion under elevated temperature and pressure or mechanical grinding, enabling the acquisition of high-purity lignin. However, this method demands harsh conditions and high energy consumption, making it challenging for industrial implementation. The biological method employs enzymes to selectively cleave the chemical linkages between lignin and carbohydrates under mild conditions, resulting in the production of ultra-high-purity lignin. Nevertheless, its industrialization has been hindered by extended processing time and inadequate separation efficacy. In contrast, the chemical method has emerged as the principal technique for industrial lignin production, owing to its high separation efficiency and relatively mild conditions.

### 3.1. Physical Method

The biomass-ethanol technology is regarded as one of the promising approaches to meet the global demand for sustainable energy. Key steps in this process include the hydrolysis and fermentation of carbohydrates and typically start with biomass comminution, which is often accompanied by fiber separation. Among existing processes for biomass comminution, steam explosion stands as the most significant, entailing heating feedstocks with saturated steam to achieve specific pressure and temperature. High-pressure steam penetrates the fiber interiors and is released from closed pores, triggering mechanical fragmentation of the fibers, followed by abrupt decompression to atmospheric pressure. Studies have shown that steam explosion pretreatment can degrade and dissolve most hemicellulose, along with a small portion of lignin and cellulose in the feedstocks. This process effectively facilitates the subsequent degradation of cellulose by cellulases, thereby functioning as a practical pretreatment strategy for straw-derived feedstocks [21]. In comparison to autohydrolysis, pulping, and other pretreatment methods, steam explosion exhibits substantial reductions in environmental impact, investment costs, and energy consumption [22]. Several researchers have proposed a process in which steam explosion is employed to enhance the efficiency of subsequent enzymatic/acid hydrolysis and fermentation [23,24,25]. In addition, the combination of steam explosion with SO_2_ pre-impregnation has also been utilized to optimize the ethanol yield from softwood [24].

### 3.2. Biological Method

For decades, considerable efforts have been dedicated to elucidating the structural characteristics of natural lignin macromolecules in plant cell walls. Prior to conducting structural analysis of lignin, it is essential to achieve efficient isolation of the polymer while preserving its native intact state. Several representative methods have been proposed for the purpose of isolating natural lignin from cell walls. For instance, milled wood lignin (MWL) was first proposed by Björkman [26]. Subsequently, Chang et al. [27] developed a cellulase-mediated lignin extraction method. Specifically, ball-milled wood powder was first subjected to cellulase treatment, followed by extraction with dioxane to remove the carbohydrate component to obtain target lignin. Sun [28] and Xu [29] devised a novel lignin isolation method integrating mild enzymatic hydrolysis and acid hydrolysis, termed enzymatic-acidolytic lignin (EAL). All these methods are applicable to investigating natural lignin in plant cell walls.

It has been found that lignin residues isolated via enzymatic hydrolysis contain high levels of carbohydrates and proteins [30,31]. The former is attributed to the limited capacity of enzymes to cleave lignin-carbohydrate bonds [32], whereas the latter is thought to originate from the enzymes employed. Recently, Tarasov [33] reviewed the studies on lignin prepared by enzymatic methods, noting that lignin residues after enzymatic hydrolysis contain carbohydrates linked via glycosidic bonds to the benzyl carbon of lignin, which is consistent with previous reports by Fukagawa et al. [34]. Such lignin-carbohydrate linkages have not been detected in samples isolated by acid hydrolysis. The acid lability of these bonds motivated Wang et al. [35] to conduct further research on the purification of lignin residues contaminated by carbohydrates.

### 3.3. Chemical Method

#### 3.3.1. Acid Hydrolysis Method

The acid hydrolysis method degrades lignocellulose by cleaving glycosidic bonds, thereby solubilizing cellulose and hemicellulose while leaving lignin as a residue. Hydrochloric acid (HCl), nitric acid (HNO_3_), phosphoric acid (H_3_PO_4_), and sulfuric acid (H_2_SO_4_) are commonly employed in this method [36], with the treatment feasible using either concentrated or dilute acids. Concentrated acid hydrolysis proceeds in two steps under atmospheric pressure and temperatures below 100 °C. In the first step, concentrated acid disrupts the crystalline structure of cellulose and solubilizes it. In the second step, dilute acid is applied to hydrolyze the glycosidic bonds, converting cellulose and hemicellulose into soluble monosaccharides [20,37], with lignin remaining as a residue. Due to the challenges associated with concentrated acid recovery and equipment corrosion, along with its inhibitory impact on downstream processing, dilute acid treatment is more favorable for industrial-scale implementation. Dilute acid hydrolysis is typically conducted at an acid concentration of 0.1~10 wt.%, which can be performed either at elevated temperatures (180~200 °C) for a short duration (~1 h) or at lower temperatures (~120 °C) for an extended duration (2~8 h) [37,38]. Among the aforementioned inorganic acids, H_2_SO_4_ is cheaper than HNO_3_ and H_3_PO_4_, making it the preferred acid catalyst for both industrial implementation and laboratory research. Brian et al. [39] first subjected corn stover to 6 M H_2_SO_4_ treatment at 150~200 °C for 1~6 h, followed by lignin extraction with formic acid and sodium hydroxide (NaOH). Juliana et al. [40] employed H_2_SO_4_ and NaOH as reagents for lignin extraction. The results indicated that single-agent treatment with H_2_SO_4_ at 121 °C for 15 min afforded a lignin yield of 50.7%. In contrast, sequential treatment with H_2_SO_4_ followed by NaOH at 121 °C for 30 min increased the yield to 74.9%. Henrique et al. [41] performed two consecutive extractions using dilute H_2_SO_4_ and NaOH sequentially, achieving an approximate lignin extraction yield of 40% from elephant grass.

#### 3.3.2. Alkaline Dissolution Method

Alkaline lignin extraction, which utilizes recyclable reagents (NaOH, potassium hydroxide, and calcium hydroxide) less corrosive than H_2_SO_4_, proceeds under milder conditions (ambient temperature). The alkaline reagents primarily interact with lignin, imparting high selectivity for lignin extraction and enhanced efficiency in its dissolution [42]. Therefore, alkaline extraction yields considerable amounts of high-purity lignin. Auxiliary methods such as hydrothermal, ultrasonic, and freezing treatment are often integrated with alkaline extraction to enhance lignin yield. Wu et al. [43] first subjected corn stover to hydrothermal processing (150 °C, 1 h), then to 1 M NaOH treatment (70 °C, 2 h), yielding 45% lignin. Hydrothermal treatment cleaves the linkages between lignin and hemicellulose, thereby facilitating alkaline extraction and significantly boosting lignin yield. Zhang et al. [44] extracted lignin by soaking bagasse in NaOH at a low temperature of 35 °C for 8 d, followed by acidification to precipitate it, yielding 88.6% lignin. Sun et al. [45] employed a hybrid process of hydrothermal treatment and 5 wt.% NaOH to extract ultrahigh-purity lignin from poplar wood, achieving a 71.8% yield at 180 °C. Higher pretreatment temperatures induce more severe lignin condensation. To mitigate this issue, Lai et al. [46] extracted lignin from larch wood by combining H_2_SO_4_, 2-naphthol, and NaOH, coupled with in situ modification using polyethylene glycol diglycidyl to suppress its repolymerization.

In addition to sodium, potassium, and calcium hydroxides, alkaline peroxides, aqueous ammonia (NH_3_·H_2_O), and ammonium hydroxide are widely employed as alkaline reagents for lignin extraction. The rapidity and uniformity of lignocellulose swelling in NH_3_·H_2_O, together with its cost-effectiveness, make it superior to other alkaline catalysts. Nahar et al. [47] pretreated corn stover pellets by soaking in 15 wt.% NH_3_·H_2_O at 60 °C for 6 h, with the maximum lignin yield reaching 17.9%. Zhao et al. [48] investigated pretreatments with NH_3_·H_2_O, hydrogen peroxide (H_2_O_2_), and an NH_3_·H_2_O-H_2_O_2_ blend, with corresponding lignin yields of 16.55%, 12.26%, and 17.45%, respectively. Oxidation and ammonolysis are the key reactions during the NH_3_·H_2_O-H_2_O_2_ blend pretreatment, in which oxidation is dominated by H_2_O_2_ and ammonolysis is driven by ammonia. H_2_O_2_ decomposes into reactive superoxide anions and hydroxyl radicals in alkaline media, which subsequently react to produce oxygen and water. A portion of the oxygen participates in lignin degradation and is incorporated into oxidized lignin.

#### 3.3.3. Organosolv Pretreatment

The organosolv has been commonly employed in the processing of various types of lignocellulose. The most frequently employed organic solvents encompass low-molecular-weight aliphatic alcohols, organic acids, and mixed solvents [49]. Presently, this method has been adopted in small-scale industrial production in countries including Canada, the Netherlands, Germany, and the United States [50]. The organosolv hinges on the solubility of lignin in specific organic solvents. During organosolv pretreatment, the main cleavage reactions in lignin cleave *α*-aryl ether bonds and *β*-aryl ether bonds, leading to lignin removal. Typically, the organosolv operates with a small amount of inorganic acid as a catalyst, whereby α-aryl ether bonds are more susceptible to cleavage than *β*-aryl ether bonds. Notably, organosolv delignification can also take place in neutral or alkaline media. In neutral media, the reaction system itself generates acidic compounds such as acetic acid and furfural as catalysts at high temperatures, assisting in the completion of delignification [51]. In alkaline media, the principal reaction involves the cleavage of *β*-aryl ether bonds [52]. With its low viscosity, ethanol can penetrate biomass more effectively and promote lignin removal. Pan et al. [53] produced organosolv pulp using ethanol as the cooking agent and H_2_SO_4_ as the catalyst, subsequently extracting lignin from the cooking liquor by adding water. Experimental findings demonstrated that the lignin produced by this method has a low molecular weight, high purity, and abundant active groups, making it applicable in chemicals such as adhesives. Generally, when low-boiling-point alcohols serve as solvents, high concentrations are required, leading to elevated costs. Teramura et al. [54] investigated the effects of low-concentration ethanol, *n*-propanol, isopropanol, *n*-butanol, and *n*-pentanol on lignin removal, finding that the hydrophobic *n*-butanol and *n*-pentanol showed superior lignin removal efficiency, with removal rates ranging from 64.7% to 74.3%. High-boiling-point alcohols have also garnered attention due to their advantages, incorporating high boiling points, low volatility, and solvent recyclability during the process. Wang et al. [55] extracted lignin using citric acid as the catalyst and 1,4-butanediol as the solvent, ascertaining that the lignin extraction rate reached up to 94%. Moreover, the yielding lignin displayed high molecular weight and thermal stability, demonstrating potential for applications in polymerization. Polyethylene glycol (PEG) has recently emerged as a promising solvent for lignin separation due to its non-toxicity, high boiling point, and ability to dissolve lignin under atmospheric pressure. It can also graft-modify lignin, thereby effectively enhancing its thermal flowability. Lignin prepared by this method demonstrates high thermoplasticity and can be applied in manufacturing high-performance composite materials [56]. Table 1 summarizes relevant research on organosolv processes in recent years and provides extraction conditions, yields, and purities of lignin from different substrates. As shown in Table 1, employing ionic liquids (ILs) with acidic groups (such as HSO_4_^−^) as catalysts in organosolv processes significantly increases both the yield and purity of the extracted lignin.

MWL is defined as the lignin isolated by extracting feedstocks with organic solvents, grinding them in a ball mill, and then further extracting with dioxane [26]. This process proceeds under mild conditions, causing minimal structure damage to lignin. Given that its chemical structure closely resembles natural lignin, MWL has become a core research subject for exploring the structural characteristics of lignin. Rencoret et al. [76] extracted MWL from Paulownia fortunei and characterized its structure via pyrolysis analysis and nuclear magnetic resonance spectroscopy. The results indicated that Paulownia fortunei lignin predominantly consists of guaiacyl and syringyl structural units, with only trace amounts of *p*-hydroxyphenyl units. Lignin monomers are primarily linked through *β*-O-4 aryl ether bonds, alongside a small proportion of *β*-*β* bonds and *β*-5 bonds.

#### 3.3.4. ILs Method

Within lignin isolation, the ILs method stands as a novel technique. ILs are typically defined as organic salts with a melting point below 100 °C, comprising bulky, asymmetric organic cations paired with either organic or inorganic anions, and are characterized by high thermal stability and superior electrochemical stability. The method can selectively cleave the linkages among cellulose, hemicellulose, and lignin, enabling the dissolution of these macromolecules and thus facilitating the isolation of lignin, which is termed ionic liquid lignin. Given their superior solubility for cellulose, hemicellulose, and lignin, ILs have established themselves as a promising class of solvents and thus garnered significant research attention in biomass pretreatment. During the isolation of lignin using the ionic liquid method, *β*-*β* linkages undergo cleavage, thereby causing structural damage to it. Its weight-average molecular weight is only two-thirds that of MWL. However, the methoxyl and phenolic hydroxyl group contents in ionic liquid lignin are comparable to those in MWL, at 15.5% and 6.7%, respectively, whereas the distribution of lignin fragments is more uniform [77]. Herein, recent research on the extraction of lignin via ILs is briefly summarized, as presented in Table 2.

**Figure 4:**
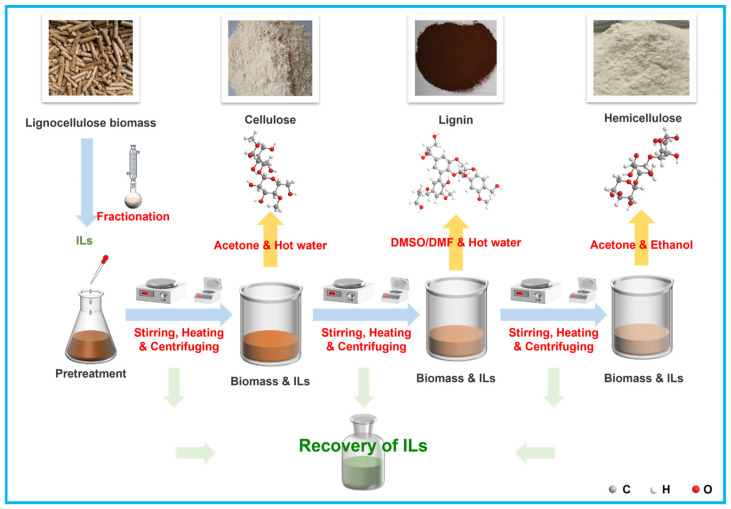
Process of pretreatment for lignocellulose [2].

Differences in the anions and cations of ILs both exert effects on lignin solubility. Pu et al. [90] investigated the influence of different anions on lignin solubility with 1-butyl-3-methylimidazolium ([BMIM]^+^) as the cation. The results demonstrated that the order of anionic influence was trifluoromethanesulfonate ([OTf]^−^) > [Cl]^−^ > [Br]^−^ > [PF_6_]^−^, indicating that the anions of ILs can affect lignin solubility. Wang et al. [91] examined the effect of cations in a series of imidazolium-based ILs on lignin extraction. The experimental findings revealed the order of lignin solubility for different cations as follows: [BMIM]^+^ > 1-hexyl-3-methylimidazolium ([HeMIM]^+^) > 1-ethyl-3-methylimidazolium ([EMIM]^+^) > 1-octyl-3-methylimidazolium ([OMIM]^+^) > 1-butyl-3-ethylimidazolium ([BEIM]^+^) > 1-butyl-3-propylimidazolium ([BPIM]^+^). They also observed that as the alkyl chain lengthens, the steric hindrance of the cations increases, thereby hindering the contact of cations and anions with lignin and reducing lignin solubility. Hou et al. [92] synthesized a series of renewable choline-amino acid-based ILs, namely [Ch][Lys], [Ch][Gly], [Ch][Ala], [Ch][Ser], [Ch][Thr], [Ch][Met], [Ch][Pro], and [Ch][Phe], through simple acid-base reactions. They determined that these 8 ILs displayed poor solubility for xylan and cellulose yet excellent solubility for lignin and emphasized that pretreatment temperature and time are crucial to delignification. The renewability, low toxicity, and ready biodegradability of these choline-amino acid-based ILs were also investigated, and these characteristics are aligned with the development concept of green chemistry [93]. Considering the high selectivity of ILs for lignin separation and the non-polluting merits of the organosolv pretreatment process, Wang et al. [63] used a hydrothermal method to separate bamboo in an ILs-ethanol-water system. The findings indicated that the lignin recovery rate reached 94.13% with a purity of 97.23%, both of which were higher than those of single-treatment processes. The detailed process of common ILs-based pretreatment for lignocellulose is illustrated in Figure 4.

## 4. Comparison of Separation Methods

The separation and extraction of lignin serve as the foundation of its structural research and practical applications. Thus, developing efficient and cost-effective separation methods is of critical importance to advancing lignin research. Up to now, efforts devoted to lignin separation research have been ongoing, but the technology still lacks maturity and thus remains restricted to laboratory-scale experiments. Although numerous separation methods are available, their variations in mechanisms, characteristics, and compatible feedstocks lead to significant differences in lignin isolation efficiency and operational costs. Therefore, it is necessary to design targeted separation methods tailored to different feedstocks and applications. Chemical separation methods boast broad application prospects due to their diverse types, high lignin yield, and high purity. Table 3 presents various chemical separation methods in terms of their applicability, advantages and disadvantages, key challenges, and main operational costs. Each method possesses its merits and drawbacks, among which the alkaline dissolution method stands out as the most mature. Currently, most of the commercially available lignin is alkali lignin, which gives rise to a critical issue of product homogeneity.

As shown in Table 3, organosolv pretreatment ranks as the most promising method for large-scale production, second only to alkaline dissolution pretreatment, given its minimal structural damage to lignin and ease of operation. The organosolv primarily employs organic solvents such as methanol, ethanol, acetic acid, and acetone to delignify by cleaving the linkages between lignin and hemicellulose [97]. However, exclusively using organic solvents for lignin extraction causes poor solubility of hemicellulose and cellulose, which in turn leads to low lignin yield and purity. Typically, acidic catalysts are incorporated during the delignification process to facilitate the cleavage of interlinkages between lignin and hemicellulose, thereby achieving significant yields of high-purity lignin [98]. Traditional acidic catalysts are predominantly composed of inorganic acids such as H_2_SO_4_ and HCl. These inorganic acids feature high toxicity, severe corrosivity towards equipment, and inferior separability and recoverability, thus impeding their large-scale industrial implementation [2,86]. ILs have been extensively employed as solvents and/or catalysts for the catalytic conversion of biomass, owing to their distinctive properties, including low melting points, excellent thermal stability, low toxicity, minimal corrosivity, high designability, and facile separability and recyclability [99]. In this context, introducing inorganic acids into ILs to fabricate acid-functionalized ILs, and employing them as catalysts in the organic solvent-mediated delignification process, holds promise for addressing the prevailing challenges encountered in organosolv pretreatment technology.

## 5. Conclusions and Outlook

As the only renewable aromatic polymer found in nature, the complex three-dimensional structure of lignin and its intrinsic propensity for condensation during separation processes constitute obstacles to the efficient isolation and valorization. Presently, lignin separation techniques primarily encompass physical, chemical, and biological methods. Physical methods exhibit high energy consumption, while biological methods operate under mild conditions yet suffer from low efficiency. Among chemical methods, acid hydrolysis demonstrates high efficiency but tends to induce lignin condensation, whereas alkaline dissolution has been widely adopted but is burdened with elevated costs and notable pollution. Organosolv and the emerging ILs method demonstrate promising application potential, given their tunable reaction conditions and minimal structural disruption to lignin. Notably, ILs, with their high tunability and superior solubility, despite their current high cost and laboratory-scale phase, emerge as a pivotal direction in future green separation technologies.

The structural characteristics of lignin are intrinsically linked to its functional applications, which is a key point to further clarify for promoting its high-value utilization. As emphasized in this review, the structural features of lignin, including its basic structural units (S/G/H units), inter-unit linkages (e.g., *β*-O-4, *β-β*, *β*-5, and 4-O-5 bonds), three-dimensional spatial configuration, molecular weight distribution, condensation degree, and the abundance of active functional groups (e.g., phenolic hydroxyl and methoxyl groups), directly regulate its functional performances. Additionally, the type and content of S/G/H units also affect the reactivity of lignin: hardwood lignin (G-S type) with high S-unit content has better solubility and processability, softwood lignin (G-type) with high G-unit content has stronger thermal stability, and herbaceous lignin (H-G-S type) with mixed S/G/H units shows versatile applicability. This intrinsic correlation between structure and function highlights the importance of efficient separation and structural preservation of lignin during the extraction process, as it directly determines the practical application value and development potential of lignin.

Looking ahead, efforts focus on developing green, efficient, and low-cost lignin separation technologies. Priority should be placed on optimizing the ILs-organic solvent hybrid system to enable lignin extraction with high yield, high purity, and minimal structural modification. Simultaneously, it is essential to intensify research into the fine structural analysis and reaction mechanisms of lignin to underpin its downstream value-added conversion. By establishing an integrated feedstocks-separation-products process system, the full-component biorefining of biomass can be advanced, thereby lengthening the industrial chain and ultimately enabling the large-scale and value-added utilization of lignin resources in the domains of materials, chemical engineering, and energy.

## Figures and Tables

**Table 1:** Methods of organosolv lignin extraction from different lignocellulosic substrates, optimal conditions, yield and purity.

Feedstocks	Extraction Agent	Extraction Conditions (Temperature, Time, Solids Loading (Solvent:Biomass))	Lignin Yield (%)	Lignin Purity (%)	Ref.
Wheat straw/Pine straw/Alfalfa	85% organic acid (70:30 peroxyformic acid:peroxyacetic acid)	Boiling temperature, 2 h, 8:1	20.4/34.0/22.7	-	[57]
Miscanthus	Ethanol (50%) + H_2_SO_4_ (1.2%, *w*/*w*)	170 °C	77.9	93.13–98.12	[58]
Mango seed husk	60% ethanol	148.41 °C, 15 min	70.2	96.18	[59]
Silver birch	Ethanol (60%) + H_2_SO_4_ (0.1%, *w*/*w*)	200 °C, 0.25 h, 10:1	86.2	-	[60]
Sawmill mixed	Ethanol (60%) + water (40%) + H_2_SO_4_ (0.25%, *v*/*v*)	175 °C, 30 min	50	-	[61]
Sugarcane bagasse	Ethanol (80%) + water (20%) + 2 mmol acidic IL ([C_4_H_8_SO_3_Hmim]HSO_4_)	200 °C, 30 min, 3:50	100	-	[62]
Wheat stalk	Ethanol (80%) + water (20%) + 6 mmol acidic IL ([BSTEA]HSO_4_)	200 °C, 30 min, 3:50	94.13	97.23	[63]
Black spruce	Ethanol (70%) + FeCl_3_·6H_2_O (5%)	180 °C, 90 min	74.0	97.0	[64]
Oil palm empty fruit brunch	1,4-dioxane, 0.3 M HCl	90 °C, 90 min	~8	89.65	[65]
Bagasse	Ethylene glycol (90%) + water (10%) + HCl	130 °C, 1 h, 10:1	60.0/	-	[66]
Wheat straw	Acetic acid (55%) + formic acid (30%) + water (15%)	105 °C, 2.5 h, 10:1	73	-	[67]
Sugarcane tops	Formic acid (85%)	125 °C, 1.5 h, 7.5:1	90.8	-	[68]
Poplar wood chips	*p*-TsOH (80%) + formaldehyde (1.5%)	80 °C, 0.5 h, 12.5:1	63.7	95.06	[69]
Sweet sorghum bagasse	Acetone (50%) + water (50%)	180 °C, 1 h, 10:1	70.44	-	[70]
Oil palm mesocarp fiber	Acetone (93%) + HCl (0.3%)	115 °C, 3 h, 10:1	70	86.8	[71]
Milled pine wood	*γ*-valerolactone/water (4:1) + 0.225 M H_2_SO_4_	160 °C, 24 h	41.5	-	[72]
Eucalyptus	*p-*TsOH (10%) + butanediol (70%) + water (20%)	130 °C, 4 h, 10:1	80	-	[73]
Poplar sawdust	*p*-TsOH (2 mol/L) in ethanol	85 °C, 5 h, 8:1	60	>99	[74]
Poplar	Ethyl acetate (50%) + 30% hydrogen peroxide (50%) + H_2_SO_4_ (1%)	Room temperature, 72 h	97.2	-	[75]

**Table 2:** Summary of recent research on lignin extraction using ILs.

Feedstocks	ILs and Solvent	Reaction Conditions	Yield/%	Ref.
Wheat straw/rice husk	Pyridinium protic ILs having clusters around the anion H_2_PO_4_	100 °C, 2 h	73	[78]
Rice husk	[PSMIM]Cl	100 °C, 6 h	78	[79]
	[PSMIM]Ace		53	
	[PSMIM]HSO_4_		11	
Eucalyptus wood	[HMIM]Cl	135 °C, 6 h	82.35	[80]
Miscanthus × giganteus	[EOA]OAc in ethanol-water	120 °C, 10 h	32.0 ± 1.5	[81]
Hybrid poplar wood powder	[AMIM]Cl in *p*-TsOH	80 °C, 15 h	86	[82]
	[AMIM]Cl in ChCl-Lac		11	
	[AMIM]Cl		0	
Eucalyptus urophylla powder	[EMIM]OAc	140 °C, 40 min	45.8 ± 2.0	[83]
Olive pomace	[TEA]HSO_4_:H_2_O (19:1 = W/W)	150 °C, 2 h	40	[84]
Rice straw/rice husk/wheat straw/sugarcane bagasse	[TEA]HSO_4_:H_2_O (4:1 = W/W)	170 °C, 30 min	80–90	[85]
		170 °C, 45 min		
Eucalyptus wood chips	[EMIM]OAc:H_2_O (17:3 = W/W) [EMIM]OAc:H_2_O (3:17 = W/W)	150 °C, 3 h	41.5 37.1	[86]
Tobacco stem	[EMIM]DEP	150 °C, 4 h	85.38	[87]
Coffee husk	[DIPEA]Ac	120 °C, 4 h	71.2	[88]
Sugarcane bagasse	[EMIM]OAc	140 °C, 2 h	90	[89]

Note: [PSMIM]Cl = 1-methyl-3-(3-sulfopropyl)imidazolium chloride; [PSMIM]Ace = 1-methyl-3-(3-sulfopropyl)imidazolium acetate; [PSMIM]HSO_4_ = 1-methyl-3-(3-sulfopropyl)imidazolium hydrogen sulfate; [HMIM]Cl = 1-hexyl-3-methylimidazolium chloride; [EOA]OAc = ethanolamine acetate; [AMIM]Cl = 1-allyl-3-methylimidazolium chloride; [EMIM]OAc = 1-ethyl-3-methylimidazolium acetate; [TEA]HSO_4_ = triethylammonium hydrogen sulfate; [EMIM]DEP = 1-ethyl-3-methylimidazolium diethyl phosphate; [DIPEA]Ac = diisopropylethylammonium acetate.

**Table 3:** Comparison of multiple commonly used chemical separation methods [2,94,95,96].

Separation Method	Feedstocks	Industrial Application	Main Operating Cost	Average Molecular Weight (kDa)	Advantages	Major Issues
Acid hydrolysis	All feedstocks	Relatively mature, mainly used in bioethanol production processes	Energy consumption, acid costs	2–6	Cellulose and hemicellulose are hydrolyzed into soluble sugars with high conversion efficiency, short reaction time and fast separation speed	Significant chemical changes in lignin; acids are corrosive
Alkaline dissolution	Hardwoods, agricultural wastes (with high lignin content), harsh reaction conditions for softwoods	Widely applied in industrial production, mainly used in pulping processes	Energy consumption, recovery of alkali and lignin from black liquor, alkali costs	2–8	Milder reaction conditions compared to acid hydrolysis; cleaves the linkages between carbohydrates and lignin, resulting in high lignin yield	Chemical changes in lignin, higher alkali cost compared to other solvents, longer treatment time required under cooling conditions, formation of non-recyclable salts
Organosolv	Softwoods, hardwoods, herbaceous plants	Small-scale production in countries such as Canada, the Netherlands, Germany, and the United States	Energy consumption, organic solvent recovery	2–10	Cleaves the linkages between lignin and hemicellulose and reduces the lignin sedimentation rate, resulting in high lignin purity and good removal efficiency	Catalysts are highly corrosive and toxic, prone to causing environmental pollution; some organic reagents are flammable and explosive
ILs	Softwoods, hardwoods, herbaceous plants	Only in the laboratory research phase	Energy consumption, ILs costs	1–8	ILs have high thermal stability and non-volatility, and can be selected and combined as needed, with good solubility and selectivity for lignin	Less chemical changes in lignin, ILs are expensive and their synthesis process is complex

## Data Availability

No new data were created or analyzed in this study. Data sharing is not applicable to this article.
